# Quality in Contemporary Surgical Nursing

**DOI:** 10.3390/clinpract14040096

**Published:** 2024-06-21

**Authors:** Georgios Vasilopoulos

**Affiliations:** Department of Nursing, University of West Attica, 12243 Athens, Greece; gvasilop@uniwa.gr

Quality in healthcare is a multidimensional issue involving various features that depend on service performance and personal assessment (Stavropoulou et al., 2022, [1]). In this Special Issue, we seek to highlight the need for continuous improvement and adaptation in healthcare. For the editorial, I chose to focus on quality of care in surgical nursing. The following article reviews the key factors that influence quality in contemporary surgical nursing, including education and continuing professional development, technology integration, person-centred care, and patient safety.

Surgical nursing constitutes one of the most challenging and critical areas of nursing care. Continuous quality improvement is essential to ensure patient safety, improve clinical outcomes, and promote health.

## 1. Education and Training

The training of surgical nurses is fundamental to the provision of quality care. Nurses must have a thorough knowledge of the procedures and techniques used at all stages of the care of the surgical patient, as well as the latest developments in their scientific field. Studies have shown that continuing education reduces error rates and increases patient confidence in nursing staff (Audet et al., 2018, [2]). In addition, collaboration with academic institutions and professional bodies to develop educational programmes and promote research in the field of surgical nursing is essential. These partnerships can help to highlight new challenges and drive the development of innovative solutions.

## 2. Technology and Innovation

Technology has undoubtedly changed the landscape of the surgical sector. Modern technologies such as patient monitoring systems, robotic surgery, and digital data collection and analysis platforms have significantly improved the quality of patient care. The integration of these technologies into the daily practice of surgical nurses enables immediate access to critical information and informed decision making. The use of advanced technologies in surgical care in combination with ERAS protocols (enhanced recovery) has been shown to reduce complications, improve patient recovery, and increase patient satisfaction (Choi et al. 2022 [3]; Reddy et al., 2023 [4]). However, it is critical that nurses in the surgical setting receive ongoing education in the use of new technologies to ensure their effective and safe implementation.

## 3. Patient-Centred Care

Patient-centred care is a cornerstone of quality in healthcare. Good communication with patients and their families, understanding their needs and expectations, and providing emotional support are fundamental elements of the person-centred approach. Patients who feel understood and emotionally supported have less anxiety and recover more quickly (Legg et al., 2014 [5]). Developing communication skills and awareness of patients’ cultural and social differences is also important. Surgical nurses need to be able to adapt to the different needs of patients and provide individualised care that responds to their particular circumstances.

## 4. Patient Safety

Patient safety is of paramount importance in surgical nursing. The implementation of strict safety protocols, the use of standards and guidelines, and the continuous evaluation and improvement of procedures are essential to prevent complications and ensure better clinical outcomes. The World Health Organization has emphasised the need for strict safety protocols in surgery to prevent complications and ensure better outcomes (WHO, 2018, [6]). Reporting and analysing incidents of errors and deviations can also help to improve safety. Surgical nurses should be encouraged to report any problems or errors without fear of repercussions so that their causes can be identified and addressed.

## 5. Conclusions

Quality in modern surgical nursing is multidimensional and requires a combination of technological innovation, continuing education, and a person-centred approach. Continuous improvement and adaptation to new challenges are essential to ensure high-quality care and patient safety. Surgical nurses must be prepared to meet the challenges of modern medical practice and promote quality and safety at every stage of the care they provide.
